# Association of Pain, Insomnia, and Depression Clusters with Cognition in Older Mexican Americans

**DOI:** 10.1093/geroni/igaf122.4072

**Published:** 2025-12-31

**Authors:** Utpol Das, Matthew Phan, Tyler Bell, Mukaila Raji, Sadaf Milani

**Affiliations:** University of Texas Medical Branch, Galveston, Texas, United States; University of Texas Medical Branch, Galveston, Texas, United States; University of California, San Diego, San Diego, California, United States; University of Texas Medical Branch, Galveston, Texas, United States; University of Texas Medical Branch, Galveston, Texas, United States

## Abstract

Older Mexican Americans—a rapidly growing segment of the U.S. population—experience high rates of co-occurring pain, depression, and insomnia, but data on how these symptom clusters affect their cognition remain limited. We examined patterns of pain-insomnia-depressive symptom clusters and their association with cognitive decline using data from the Hispanic Established Populations for the Epidemiologic Study of the Elderly (2010-2016), a study of older Mexican Americans in the Southwestern United States. Latent class analysis identified clusters based on pain on weight-bearing, depressive symptoms, and insomnia symptoms. Linear mixed-effects models examined the association between different patterns of symptom clusters and cognitive decline, measured by the Mini-Mental State Examination. Three latent classes were identified (n = 935): pain only (66.5%), pain and elevated depressive symptoms, (21.0%), and pain, elevated depressive symptoms, and insomnia (12.5%). The pain and elevated depressive symptoms cluster (β=-3.72, p < 0.001) and the pain, depressive symptoms, and insomnia cluster (β=-2.33, p < 0.001) were associated with worse baseline cognition compared to the pain only cluster. Education was a significant effect modifier: in stratified analyses, similar findings were observed for those with 0 and 1-6 years of education. Among individuals with ≥7 years of education, those in the pain and elevated depressive symptoms cluster had greater cognitive decline over time compared to the pain only cluster (β=-0.65, p = 0.039). Our findings highlight the need for targeted interventions addressing co-occurring conditions to mitigate cognitive decline among older Mexican Americans. Education appears to modify this association, suggesting a role in cognitive reserve and warranting consideration in intervention design.

